# Microbes and colorectal cancer: is there a relationship?

**DOI:** 10.3747/co.v16i4.472

**Published:** 2009-08

**Authors:** J.M. Uronis, C. Jobin

**Affiliations:** * Department of Medicine and Center for GI Biology and Disease, University of North Carolina at Chapel Hill, Chapel Hill, NC, U.S.A; † Department of Pharmacology, University of North Carolina at Chapel Hill, Chapel Hill, NC, U.S.A

**Keywords:** Colitis, colorectal cancer, inflammatory bowel disease, Nod-like receptor, Toll-like receptor, bacteria

The human colon plays host to as many as 15,000–36,000 bacterial species, amounting to more than 100 trillion bacteria 1,2. The microbiota and their associated prokaryotic genome is an integral part of the host and uniquely contributes to various biologic processes such as maturation and development of the mucosal immune system, metabolic capacity, and intestinal epithelial cell proliferation and differentiation 3. An international effort is currently underway to catalogue the repertoire of microorganisms present in the intestines of healthy humans and of those with pathologic conditions. The human microbiome project—for which the U.S. National Institutes of Health has contributed more than $110 million—is aiming to determine the structure of the microbial community associated with the human body and the functions thereby served in health and disease 3,4.

Arguably, mapping and comparing the composition of the microbial community harboured by healthy and disease-affected humans represents the next frontier in microbiology and medicine. Although this field of research is still in its infancy, the link between the microbiota and development of inflammatory bowel diseases (ibds) has been established in animal models and human patients alike. Countless studies have established that an improper innate or adaptive host response to microbial constituents leads to unrestricted activation of immune cells—including T-lymphocyte effector cells—and development of chronic inflammation 5.

One of the greatest risks assumed by individuals with ibd is a heightened susceptibility to colorectal cancer (crc). Epidemiology studies indicate that the duration and severity of chronic colitis are important risk factors for crc 6–8. Because the microbiota play an essential role in the development of ulcerative colitis and Crohn disease (the two main manifestations of ibd), a close look at the relationship between bacteria and crc is a must.

## EVIDENCE THAT MICROBES ARE INVOLVED IN CRC

A tantalizing observation linking bacteria with the development of crc was made more than a decade ago through germ-free technology, in which mice are born and maintained in the absence of microorganisms. Dove *et al.* 9 observed that tumour development in germ-free *Apc^Min^* (multiple intestinal neoplasia allele of the adenomatous polyposis coli gene) mice, which serve as a model for human familial adenomatous polyposis, was halved as compared with that in mice housed under specific pathogen-free conditions.

More recent findings implicating the microbiota as key players in development of crc came with the discovery of an important communication system between humans and bacteria. Innate bacteria-sensing receptors such as the Toll-like receptor (tlr) and the Nod-like receptor alert the host to the presence of bacteria 10. Interestingly, deletion of the myeloid differentiation factor 88 (myd88) adapter protein, a key mediator of the tlr/interleukin 1 (il-1) signalling pathway, attenuated polyp formation in the *Apc^Min^* mouse model 11. These findings support the idea that bacteria use tlr-mediated signalling components such as myd88 to promote the development of crc. However, because the pro-inflammatory cytokine il-1β uses myd88 to signal downstream of its receptor, a formal demonstration that bacterial signalling factors participate in tumour progression is still needed in this model.

## EVIDENCE THAT MICROBES ARE INVOLVED IN COLITIS-ASSOCIATED COLON CANCER

A significant risk associated with ibd is the development of crc, a collective pathophysiologic event called colitis-associated colorectal cancer (cac). The risk of cac developing in individuals affected with ulcerative colitis for 30 years or more has been evaluated at 7.6%–18% 8. Mouse models of cac have been integral to advancing an understanding of the effect of the gut microbiota on chronic colitis and cancer development.

In the most widely used mouse model of cac, administration of the colon-specific carcinogen azoxymethane (aom) induces initiating genetic mutations in the wnt/ctnnb1 (β-catenin) pathway; subsequently, administration of dextran sodium sulfate (dss) disrupts the colonic epithelium, inducing chronic colitis 12. Work with this model showed that deletion of *Tlr4* decreased tumour formation, suggesting that bacteria use the receptor to promote colorectal carcinogenesis 13. In contrast, deletion of *Nod1* predisposes to aom–dss-induced cac 14. Because tlr4 and nod1 recognize different microbial spectra (extracellular and intracellular respectively), these apparently opposite functions could highlight specific roles for microorganisms in protecting against or promoting cancer development. Clearly, establishing the composition of the microbial community present in a healthy as compared with a cancer-prone intestine may provide insight into the role of bacteria in tumorigenesis.

An intriguing observation from studies using aom–dss-induced cac in *Tlr4*^−/−^ mice is that attenuation of colorectal tumorigenesis occurs without a clear concomitant reduction in the severity of inflammation. Interestingly, a similar dissociation between inflammation and tumour development was recently observed in aom–dss-exposed *Il6*^−/−^ mice 15. In that study, the authors demonstrated a strong reduction in tumour development in *Il6*^−^*^/^*^−^ mice despite inflammation being significantly augmented in those mice as compared with wild-type mice. These findings contrast with the epidemiology data, which indicate that severity and duration of chronic colitis directly correlates with crc risk 7.

The inherent difficulty encountered with the aom–dss model in the investigation of inflammation-driven tumorigenesis lies in its inability to uncouple processes elicited by acute wound-healing from those of a chronic inflammatory response. This difficulty may highlight a limitation of the aom–dss model in the study of bacterial/tlr signalling in cac, because this pathway protects against intestinal injury induced by dss 16. Indeed, although dss-induced intestinal injury is worsened in *Myd88*^−^*^/^*^−^ mice, spontaneous colitis observed in *Il10*^−/−^ mice is attenuated in *Il10*^−/−^; *Myd88*^−/−^ mice 17,18.

To avoid use of a chronic injury model, researchers have substituted the spontaneous *Il10*^−^*^/^*^−^ mouse model of intestinal inflammation for the aom–dss model. Work using a combination of this aom–*Il10*^−^*^/^*^−^ model of cac and gnotobiotic techniques recently identified a clear role for the microbiota in the development and progression of cac 19. Whereas wild-type mice treated with aom develop rare, low-grade colonic adenomas, *Il10*^−/−^ mice with normal intestinal microbiota show a dramatic increase in crc susceptibility, which directly correlates with the severity of intestinal inflammation. Conversely, aom-treated *Il10*^−/−^; *Myd88*^−/−^ mice and aom-treated *Il10*^−/−^ mice housed under germfree conditions fail to develop cac 19. These findings highlight the essential role that the intestinal microbiota play in the development of cac.

From the foregoing studies, it has become clear that the microbiota affect colorectal tumour development and progression. Certain innate sensors, such as nod1, appear to prevent tumour development; others, such as tlr4 and myd88, promote carcinogenesis. Further investigation will be required to elucidate the nature of these bacterial-mediated tumour-suppressing and -promoting signals. Identification of the microbial communities associated with these carcinogenic events will be equally important. Finally, key observations made using the aom–*Il10*^−/−^ model have to be validated in other animal models of cac before novel paradigms can be established.

Although identification of the microbiota as an essential factor in colorectal carcinogenesis adds complexity to the pathophysiology of this disease, this initiative may represent a novel opportunity for therapeutic intervention.
